# Structural basis of the phosphoramidate N-benzimidazole group’s influence on modified primer extension efficiency by Taq DNA polymerase

**DOI:** 10.18699/vjgb-25-112

**Published:** 2025-12

**Authors:** A.A. Berdugin, V.M. Golyshev, A.A. Lomzov

**Affiliations:** Institute of Chemical Biology and Fundamental Medicine of the Siberian Branch of the Russian Academy of Sciences, Novosibirsk, Russia Novosibirsk State University, Novosibirsk, Russia; Institute of Chemical Biology and Fundamental Medicine of the Siberian Branch of the Russian Academy of Sciences, Novosibirsk, Russia Novosibirsk State University, Novosibirsk, Russia; Institute of Chemical Biology and Fundamental Medicine of the Siberian Branch of the Russian Academy of Sciences, Novosibirsk, Russia Novosibirsk State University, Novosibirsk, Russia

**Keywords:** N-benzimidazole oligonucleotides, PABAO, molecular dynamics, structure, Taq DNA polymerase, molecular diagnostics, N-бензимидазольные олигонуклеотиды, ФАО, молекулярная динамика, структура, Taq ДНК-полимераза, молекулярная диагностика

## Abstract

We recently proposed a novel class of nucleic acid derivatives – phosphoramidate benzoazole oligonucleotides (PABAOs). In these compounds, one of the non-bridging oxygen atoms is replaced by a phosphoramidate N-benzoazole group, such as benzimidazole, dimethylbenzimidazole, benzoxazole, or benzothiazole. Studies of the properties of these derivatives have shown that their use in PCR enhances the specificity and selectivity of the analysis. The study investigates the effect of phosphoramide N-benzimidazole modification of DNA primers on their elongation by Taq DNA polymerase using molecular dynamics simulations. We examined perfectly matched primer-template complexes with modifications at positions one through six from the 3’-end of the primer. Prior experimental work demonstrated that the degree of elongation suppression depends on the modification position: the closer to the 3’-end, the stronger the inhibition, with maximal suppression observed for the first position, especially in mismatched complexes. Furthermore, incomplete elongation products were experimentally observed for primers modified at the fourth position. Our molecular dynamics simulations and subsequent analysis revealed the molecular mechanisms underlying the interaction of modified primers with the enzyme. These include steric hindrance that impedes polymerase progression along the modified strand and local distortions in the DNA structure, which explain the experimentally observed trends. We established that both different stereoisomers of the phosphoramidate groups and conformers of the phosphoramidate N-benzimidazole moiety differentially affect the structure of the enzyme-substrate complex and the efficiency of Taq DNA polymerase interaction with the modified DNA complex. Modification of the first and second internucleoside phosphate from the 3’-end of the primer causes the most significant perturbation to the structure of the protein-nucleic acid complex. When the modification is located at the fourth phosphate group, the N-benzimidazole moiety occupies a specific pocket of the enzyme. These findings provide a foundation for the rational design of specific DNA primers bearing modified N-benzimidazole moieties with tailored properties for use in PCR diagnostics.

## Abstract (ru)

Недавно нами был предложен новый класс производных нуклеиновых кислот – фосфорамидные бензоазольные олигонуклеотиды. В них один из немостиковых атомов кислорода замещен на фосфорамидную N-бензоазольную группу: бензимидазольную, диметилбензимидазольную, бензоксазольную или бензотиазольную. Изучение свойств таких производных показало, что их применение в ПЦР увеличивает специфичность и селективность анализа. Данное исследование посвящено изучению влияния фосфорамидной N-бензимидазольной модификации ДНК-праймеров на эффективность их удлинения Taq ДНК-полимеразой при помощи метода молекулярной динамики. Мы рассматривали совершенные комплексы нуклеиновых кислот с модификациями в положениях с первого по шестое считая от 3’-конца праймера. Ранее было показано, что степень подавления элонгации зависит от положения модификации: чем ближе к 3’-концу, тем сильнее ингибирование, а максимальное подавление наблюдается при модификации в первом положении, особенно в несовершенных комплексах. Кроме того, в экспериментах наблюдались продукты неполного удлинения праймеров с модификацией в четвертом положении. Проведенные компьютерное моделирование и анализ позволили выявить молекулярные механизмы взаимодействия модифицированных праймеров с ферментом, включая стерические препятствия для продвижения полимеразы по модифицированной цепи и локальные нарушения структуры ДНК, которые объясняют наблюдаемые экспериментально закономерности. Установлено, что как различные стереоизомеры фосфорамидных групп, так и конформеры фосфорамидной N-бензимидазольной группы по-разному влияют на структуру фермент-субстратного комплекса и эффективность взаимодействия Taq ДНК-полимеразы с модифицированным ДНК комплексом. Модификация первого и второго межнуклеозидного фосфатного остатка с 3’-конца праймера в наибольшей степени возмущает структуру белково-нуклеинового комплекса, а при расположении модификации в четвертом фосфатном остатке N-бензимидазольная модификация располагается в кармане фермента. Полученные результаты открывают перспективы для рационального конструирования специфичных, обладающими заранее заданными свойствами ДНК праймеров с модифицированными N-бензимидазольными межнуклеотидными звеньями для использования в ПЦР диагностике.

## Introduction

DNA-dependent DNA polymerase I from the bacterium Thermus aquaticus (Taq DNA polymerase) is a widely used enzyme for nucleic acid amplification by the polymerase chain reaction (PCR) in various applications. It possesses DNA polymerase and 5′→3′ exonuclease activities but lacks proofreading 3′→5′ exonuclease activity (Terpe, 2013). This enzyme is widely used for the detection of nucleic acids (NA) and single-nucleotide variants (point mutations) in diagnostic applications for various diseases, using diverse PCR-based methods such as real-time PCR, allele-specific PCR, and digital PCR (Kalendar et al., 2022; Starza et al., 2022). Allele-specific PCR is based on the inhibition of primer elongation when primers form duplexes with the template strand containing one or more mismatches at or near the 3′-end of the primer (Rejali et al., 2018). Often, a single nucleotide substitution that disrupts full complementarity between the primer and the DNA template does not provide sufficient specificity for polymorphism detection. To enhance specificity, additional single-nucleotide mismatches and/or structural modifications are introduced into the primer. These modifications can be incorporated either into the nucleobase or into the ribose-phosphate backbone and are typically positioned near the 3′-end of the primer (Kutyavin, 2011; Ishige et al., 2018; Chubarov et al., 2023). In particular, substitution of the non-bridging oxygen atom in the phosphodiester backbone affects both the thermodynamic stability of the primer–template duplex and the coordination of the terminal 3′-OH group within the enzyme’s active site. For example, incorporation of a phosphorothioate modification at the terminal or penultimate internucleotide phosphate linkage from the 3′-end of the primer results in only a modest reduction in elongation efficiency (5–15 %) while simultaneously enhancing amplification specificity (Di Giusto, King, 2003). Introduction of phosphoryl guanidine modifications into primer structures likewise alters the efficiency and selectivity of target nucleic acid sequence detection (Chubarov et al., 2020).

**Fig. 1.**
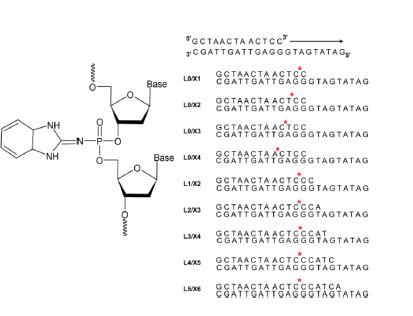
Structure of a dinucleotide step of phosphoramidate benzazole oligonucleotides containing an N-benzimidazole group and the model systems used in this study. The position of the phosphoramidate N-benzimidazole group is indicated by a red asterisk

Recently, a novel class of nucleic acid derivatives, phosphoramidate benzazole oligonucleotides (PABAOs), was developed at the Institute of Chemical Biology and Fundamental Medicine SB RAS (Vasilyeva et al., 2023). In PABAOs, the non-bridging oxygen atom of the phosphate moiety is substituted by an N-benzazole group (N-benzimidazole, Nbenzoxazole, or N-benzothiazole) (Fig. 1). PABAOs can be synthesized using standard automated solid-phase phosphoramidite chemistry.

To date, the physicochemical properties of several N-benzazole derivatives of NA have been investigated (Golyshev et al., 2024; Yushin et al., 2024; Novgorodtseva et al., 2025) and their potential use as primers in PCR, including allele-specific PCR, has been shown (Chubarov et al., 2024). We have examined the elongation efficiency of 13-mer primers containing an N-benzimidazole modification on a 22-mer DNA template using Taq DNA polymerase (Golyshev et al., 2025). When the modification is introduced at the first or second internucleotide phosphate from the 3′-end of the primer in perfectly matched duplexes, full-length extension occurs with an efficiency of approximately 50 %. In contrast, for duplexes containing a single-nucleotide mismatch at the penultimate base pair from the 3′-end of the primer, the yield of full-length product is markedly reduced. Incorporation of the modification at the third position typically results in the smallest decrease in full-length product yield among the studied positions. Furthermore, for all perfectly matched duplexes bearing the modification, a distinct aborted elongation product was consistently observed, corresponding to a partially elongated primer in which the modification was at the fourth position from the 3′-end.

In this work, we used molecular dynamics (MD) simulations to elucidate the experimental patterns of PABAO primer elongation by Taq DNA polymerase. Our study focused on how the phosphoramidate N-benzimidazole group, positioned at various sites along the primer, affects the structure and dynamics of the enzyme–substrate complex. To this end, we constructed molecular models and carried out MD simulations of both the native (unmodified) and a series of modified nucleic acid substrates containing the N-benzimidazole modification at the 1st through 6th internucleotide phosphate positions from the 3′-end of the primer, as well as their complexes with Taq DNA polymerase. The simulation results correlate well with experimental data and provide a mechanistic explanation for the effects observed in vitro.

##  Methods

Model building. The structure of the Taq polymerase–DNA complex was constructed based on the experimentally determined crystal structure with PDB ID: 1QTM as follows. The protein coordinates, including the bound nucleoside triphosphate (dNTP) and magnesium ions, were retained from this structure. The DNA complex of the template strand with the primer was modeled by building a protein–nucleic acid complex using AlphaFold3 software (Abramson et al., 2024). As input for these calculations, we provided the amino acid sequence of Thermus aquaticus DNA polymerase I (UniProt ID: P19821), along with the nucleotide sequences of the DNA template and either the unextended or partially extended primers, an incoming deoxyribonucleoside triphosphate (dNTP), and two Mg2+ ions in catalytic site. The resulting AlphaFold3-predicted structure was then superimposed onto the experimentally determined structure 1QTM by aligning the protein backbone based on Cα atoms of equivalent residues. Subsequently, the native nucleic acid components in the 1QTM structure were replaced with the DNA duplexes generated by AlphaFold3. For each constructed model, the original dNTP was substituted with the nucleotide triphosphate complementary to the base in template at the active site, ensuring correct base pairing for the elongation step under investigation.

Since the N-benzimidazole modification generally requires additional space for proper geometric accommodation within the DNA/Taq polymerase complex, we employed amino acid side-chain rotamer libraries (Shapovalov, Dunbrack, 2011) implemented in UCSF Chimera (Pettersen et al., 2004) to minimize van der Waals clashes between protein atoms and bulky modification.Partial atomic charges for amino acid residues in each complex were assigned using the pdb2pqr software (version 3.7.1) (Unni et al., 2011). The pH was set to 8.3 to match the experimental primer extension conditions (Golyshev et al., 2025). As a result, certain complexes exhibited differences in the protonation states of specific charged residues. Out of the 36 modeled complexes, seven displayed distinct protonation patterns. In the complexes L0/X2/R1, L0/X2/R2, L0/X3/ R1, L0/X3/R2, and L1/X2/R2 (notation defined below), the residues LYS540, ASP610, LYS663, and ASP785 were found in their protonated forms. In the complexes L0/X4/R1 and L0/X4/R2, the residues LYS663, LYS762, and GLU786 were also protonated.The primer/template complexes were obtained from the protein–nucleic acid complex by removing all residues except those belonging to the DNA strands.

Molecular dynamics simulation. Structural investigations of complexes formed between native or modified DNA and Taq DNA polymerase were carried out using molecular dynamics (MD) simulations and subsequent analysis with the AMBER20 software package (Case et al., 2020). Simulations were performed using parallel computing on both central processing units (CPUs) and graphics processing units (GPUs) with CUDA architecture. All MD calculations employed the ff19SB force field (Tian et al., 2020) for Taq polymerase, the OL21 force field (Zgarbová et al., 2021) for native DNA, and gaff2 parameters for the N-benzimidazolemodified phosphate residues. Parameters for magnesium and sodium ions were taken from (Li Z. et al., 2020). These force fields represent the most up-to-date and rigorously validated options currently recommended by the AMBER developers for reliable biomolecular simulations. Parameters for the deoxyribonucleoside triphosphates (dNTPs) were adopted from (Meagher et al., 2003), which remain the only published and widely accepted dNTP parameters compatible with the AMBER force field family.

MD simulation protocol. Initial models were first relaxed in implicit solvent (saltcon = 0.10 M, igb = 1, T = 1 K) using the conjugate gradient method for 2,500 steps. The systems were then solvated in an octahedral box of OPC water molecules (Izadi et al., 2014), with a minimum distance of 14 Å between any solute atom and the box boundary. Sodium ions (Na+) were added to neutralize the total charge of the periodic cell. Subsequently, the solvated systems underwent restrained energy minimization for 10,000 steps (with the first 200 steps performed using the steepest descent algorithm), applying positional restraints of 1.0 kcal/(mol·Å²) on all complex’ heavy atoms to prevent structural distortion during initial solvent relaxation. Following minimization, the systems were gradually heated from 0 to 300 K over 2 ns under constant volume (NVT ensemble), using Langevin dynamics for temperature control (ntt = 3, gamma_ln = 1.0). Pressure was then equilibrated to 1 atm over an additional 1 ns using a Monte Carlo barostat (NPT ensemble). A final unrestrained energy minimization was performed for 10,000 steps (first 200 steps: steepest descent) to remove any residual clashes after equilibration. A time step of 2 fs was used throughout, with bonds involving hydrogen atoms constrained via the SHAKE algorithm. And at the final stage, MD simulation was carried out for 100 ns with parameters similar to the heating stage, but without imposing positional restrictions on the atoms of the model system.

The MD simulation trajectories were analyzed using the cpptraj module from the AMBER20 package (Roe, Cheatham, 2013). For each trajectory, the 10 most representative structures were identified through hierarchical clustering analysis, using the average-linkage algorithm and root-mean-square deviation (RMSD) of backbone atoms as the distance metric

Molecular graphics were prepared using UCSF Chimera version 1.15 (Pettersen et al., 2004).

## Results

**Selection and construction of molecular models**

The structural and dynamic properties of PABAO complexes with Taq DNA polymerase were investigated using a comprehensive set of model systems. We employed the DNA complex formed by the primer 5′-GCTAACTAACTCC-3′ and the template strand 5′-GATATGATGGGAGTTAGTTAGC-3′, which was previously characterized in our experimental study of modified primer elongation efficiency (Golyshev et al., 2025). It has been shown that the introduction of benzoazole modifications at various positions of the primer affects the efficiency and specificity of its extension. As part of this work, MD modeling of a set of protein-nucleic acid complexes, as well as individual DNA complexes, was carried out. Both native DNA complexes and complexes containing N-benzimidazole modifications at the internucleotide phosphate groups from the 1st to the 6th position from the 3′-end of the primer were considered. To evaluate the effect of primer elongation and to obtain more reliable insights, we analyzed oligonucleotide complexes containing unextended primers with N-benzimidazole modifications positioned at 1 through 4 internucleotide phosphate from the 3′-end of the primer. In addition, we examined systems in which the primer initially bearing the N-benzimidazole modification at the first position was extended by 1 to 5 nucleotides. Following such elongation, the modification was at positions 2 through 6 relative to the new 3′-end of the primer. The sequences of the model oligonucleotide complexes and their corresponding nomenclature are provided in Figure 1

Model construction was carried out based on the crystal structure with Protein Data Bank identifier (PDB ID) 1QTM, as described in the Methods section. This structure represents a fragment of Thermus aquaticus DNA polymerase I in its closed conformation, bound to a dideoxyribonucleoside triphosphate (ddNTP) and Mg2+, and lacking exonuclease domain. The modification was introduced into the primer by replacing the native phosphate group with a phosphoramidate bearing an N-benzimidazole moiety (Fig. 1). Both stereoisomers of the phosphoramidate linkage (Sp and Rp) were considered in our study

**Fig. 2.**
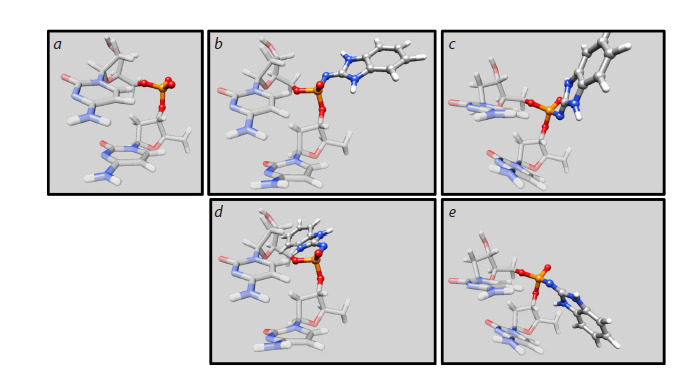
Spatial structure of DNA dinucleotide steps: native (a) and modified for the studied stereoisomers and conformers (b) R1, (c) S1, (d) R2 and (e) S2.

Analysis of the constructed molecular models of modified DNA in complex with Taq polymerase revealed that, for each phosphoramidate stereoisomer (Sp and Rp), the N-benzimidazole group can adopt two distinct orientations. These orientations correspond to the dihedral angle OP–P–N–C (where OP is the bridging oxygen, P is the phosphorus atom, N is the benzimidazole nitrogen, and C is the adjacent carbon in the heterocycle) of approximately –100 or +100°. Preliminary molecular dynamics simulations of the protein–nucleic acid complexes indicated that no transitions occurred between these two orientations of the N-benzimidazole group during the simulation timescale. Therefore, we explicitly considered both conformers (rotamers). For the model DNA complexes, we adopted the following nomenclature: Li/Xj/Rk and Li/Xj/Sk, where i = 0–5 denotes the number of nucleotides by which the primer has been elongated, j = 1–6 indicates the position of the internucleotide phosphate (counting from the 3′-end of the primer) at which the N-benzimidazole modification is introduced, k = 1, 2 specifies the rotameric conformation of the benzimidazole group for each phosphoramidate stereoisomer. For the rotamers R1 and S2, the dihedral angle defined by the atoms OP2–P–N–C (for the Rp isomer) or OP1–P–N–C (for the Sp isomer) was approximately –100°. In contrast, for rotamers R2 and S1, the corresponding dihedral angle adopted a value of approximately +100°. In these configurations, the spatial orientation of the benzoazole ring in the R1 and S1 rotamers directs the modified group away from the major groove of the DNA duplex, whereas in the R2 and S2 rotamers, the benzoazole ring is oriented toward the minor groove (Fig. 2). For modeling, 36 complexes were built with modified DNA and three with native DNA – non-extended and two extended by 3 and 5 nt (L0, L3 and L5). Simulations were also carried out for all DNA from these models

During the construction of the protein–DNA complexes L3/X4/S2, L0/X4/S2, L0/X2/S2, and L4/X5/S2, significant steric clashes were observed between the N-benzimidazole– modified DNA residue and the surrounding protein residues. In these cases, either the initial models were too distorted to proceed with stable MD simulations, or during the early stages of simulation (within the first few nanoseconds), the S2 rotamer spontaneously converted to the S1 conformation to relieve the clashes. To enable simulations with the S2 rotamer, we started from the relaxed structure of the corresponding S1 complex and performed 25 ns of restrained MD simulation in which a flat-bottom harmonic potential was applied to the dihedral angle OP1–P–N–C to gradually drive the system toward the S2 conformation (during the first 0.2 ns, the force constant of the restraint was linearly increased from 0 to 1, while the flat-bottom potential was defined with “walls” at –130.0 to –125.0° and –115.0 to –110.0°, the force constant for the restraining potential was set to 200.0 kcal/mol/rad). Following this restrained relaxation, the rotamer of the modified residue adopted the desired S2 conformation within the protein–DNA complex. Subsequently, a 100-ns unrestrained production MD trajectory was generated from this stabilized structure. This trajectory was analyzed using the same protocols applied to all other simulated systems.

**Conformational flexibility analysis**

*Stability of the protein–nucleic acid complex*

During MD simulations, the protein structure in certain models underwent noticeable conformational rearrangements, as evidenced by a pronounced increase in root-mean-square deviation (RMSD) values for the protein backbone (Fig. S1)1. In these trajectories, the RMSD exhibited considerable fluctuations during the first 50 ns, indicating incomplete equilibration. To ensure robust and reliable analysis, we extended the simulations of these specific complexes by an additional 50 ns beyond the initial 100-ns run, allowing the systems to reach an equilibrium. The RMSD profiles for the full 150-ns trajectories are shown in Figure S1. For all subsequent structural and dynamic analyses, we used only the final 50 ns


Supplementary Materials are available in the online version of the paper: https://vavilov.elpub.ru/jour/manager/files/Suppl_Berdyugin_Engl_29_7.pdf


Analysis of the MD trajectories revealed that the singlestranded region of the template strand exhibited high conformational flexibility and, as expected, did not adopt any stable or preferred conformation during the simulations. Due to its intrinsic disorder and lack of defined structural features, this single-stranded segment was excluded from further structural analysis. Figure S2 shows the RMSD profiles along the trajectories for all studied complexes. It is evident that, over the 50-ns analysis segment, all structures remain stable, as indicated by the plateauing of RMSD values after an initial brief increase during the first 1–5 ns. The average RMSD value across all analyzed complexes is 2.63 ± 0.29 Å, with a mean standard deviation along the trajectory of 0.39 ± 0.11 Å.

*Protein structural stability*

To assess structural changes in the protein during MD simulations, RMSD time profiles were calculated for the protein Cα atoms over the last 50 ns of each trajectory, using the first frame of the respective analysis segment as the reference structure (Fig. S3). The presented data clearly indicate that, following initial relaxation during the first 50 ns, the protein structure remains highly stable in all modeled complexes.

The analysis of RMSD distributions across the trajectories, presented in Figure S4, shows that RMSD values remain within a narrow range, below 3.5 Å, and the distributions themselves are relatively sharp, confirming the high conformational stability of the protein throughout the simulations. The presence of multiple peaks in some RMSD distributions indicates that the system samples several distinct yet closely related conformational substates during the simulation. This observation is corroborated by the subsequent hierarchical cluster analysis (see below), which identifies multiple populated clusters corresponding to these substates. Importantly, the structural differences between these clusters are minor.

*Stability of the DNA structure within the complex*

To assess DNA structural changes during MD simulations, we calculated the RMSD over the last 50 ns of each trajectory, using the first frame of this segment as the reference structure (Fig. S5). For this analysis, we considered two distinct representations of the nucleic acid component: the duplex region only and the full DNA construct, including the single-stranded 5′-overhang of the template strand. This is attributed to the high conformational flexibility of the singlestranded overhang. As shown in the data, the duplex region of the DNA remains highly stable in all trajectories after the initial 50 ns. The RMSD analysis along the trajectories for DNA in complex with the protein performed both including and excluding the single-stranded template overhang revealed a significant difference in the average RMSD values and their standard deviations (averaged across all models). When the single-stranded overhang was included, the mean RMSD was 3.46 ± 0.97 Å, with a trajectory-wise standard deviation of 0.84 ± 0.31 Å. In contrast, when only the duplex region (primer–template hybrid) was considered, the mean RMSD dropped significantly to 1.97 ± 0.77 Å, with a much lower standard deviation of 0.39 ± 0.12 Å. Thus, to ensure a reliable and meaningful structural analysis, we excluded the singlestranded DNA segment from our evaluations, as it adopted highly variable conformations along the MD trajectories and did not exhibit a stable or functionally relevant orientation within the complex

*Stability of the structure for simulated free DNA*

RMSD analysis of DNA trajectories in the absence of protein revealed significantly higher conformational mobility compared to the DNA within the Taq polymerase complex (Fig. S6). For the full DNA construct (including the singlestranded overhang), the average RMSD and its standard deviation (averaged across all models) were 5.11 ± 1.72 and 1.29 ± 0.61 Å, respectively. When the single-stranded region was excluded, these values decreased to 2.45 ± 0.41 and 0.50 ± 0.12 Å. These results clearly demonstrate that Taq polymerase substantially restricts the conformational flexibility of both the duplex and single-stranded regions of DNA upon complex formation. Moreover, the greater spread in RMSD values (evidenced by higher standard deviations) for free DNA indicates a broader ensemble of sampled conformations, whereas the protein-bound DNA adopts a more constrained and homogeneous structural state.

*Analysis of protein, DNA, and protein–nucleic acid complex structures*

To evaluate the impact of the N-benzimidazole modification on protein conformation, we calculated pairwise RMSD values between Cα atoms of the most representative structures (i. e., cluster centroids) extracted from the last 50 ns of each MD trajectory via hierarchical clustering. These RMSD values were used to construct a two-dimensional heatmap (Fig. S7), which visualizes structural similarities and differences across all simulated complexes. The analysis revealed that the average RMSD between native and modified complexes is very similar, with a mean value of ~2.60 Å, indicating that the overall protein fold is largely preserved regardless of the presence, position, or stereochemistry of the modification. However, when comparing individual modified systems, spanning different modification positions (X1–X6), stereoisomers (Rp/Sp), and rotamers (R1/R2, S1/S2), the pairwise RMSD values exhibit a broader range, from 1.31 to 4.37 Å. Notably, the average RMSD of each structure relative to all others falls within a relatively narrow interval of 2.33–3.26 Å (Table S1), confirming that all modeled complexes adopt globally similar conformations. The average RMSD values for each modification position, averaged over both stereoisomers and rotamers follow the trend: X1 < X2 < X6 < X4 < X3 <; X5. This ordering indicates that modifications at positions X3 and X5 induce the largest structural perturbations in Taq polymerase, whereas modifications near the 3′-terminus (X1, X2) are best accommodated with minimal impact on the protein conformation. Furthermore, when RMSD values are averaged across all modification positions for each rotamer/stereoisomer type, the following trend emerges: S1 > R1 > R2 > S2. This sequence correlates directly with the spatial orientation of the N-benzimidazole group relative to the DNA duplex, the benzimidazole moiety toward the major groove leading to greater steric interference with polymerase residues.

**Fig. 3.**
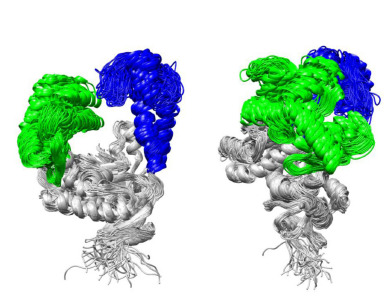
Superposition of the most representative protein structures from the MD trajectories of all studied complexes, obtained by hierarchical clustering. The palm domain is shown in gray, the thumb domain in blue, and the fingers domain in green. Protein structures were aligned based on the palm domain to highlight conformational differences in the mobile domains. The panel on the right shows the same superposition rotated by 90° around the vertical axis relative to the left panel, providing a side view of domain arrangements.

Comparison of the most representative structures from the MD trajectories across all model complexes reveals that structural differences are primarily localized to the fingers and thumb domains, while the palm domain remains remarkably stable in all systems (Fig. 3). Additionally, the N-terminal region of the protein exhibits high conformational flexibility. Such variations are associated both with the conformational mobility of the thumb and fingers domains and with the effect of modification on their arrangement.

*Structure of DNA*

It is well established that nucleic acid (NA) substrates undergo significant conformational rearrangements upon binding to DNA polymerases compared to their solutionstate structures (Vinogradova, Pyshnyi, 2010). Key structural changes commonly observed in experimentally determined polymerase–DNA complexes include: sugar pucker conformational shifts, narrowing of the minor groove, and induction of a pronounced bend in the DNA duplex at the active site. To characterize these effects in our systems, we compared the structures of the DNA substrate in the free state (i. e., without protein) and in complex with Taq DNA polymerase, using the most representative conformations identified by hierarchical clustering of the MD trajectories. RMSDs between the duplex regions of the free and proteinbound DNA structures were calculated for all combinations of stereoisomers (Rp and Sp), rotamers (R1/R2 and S1/S2), and extension states (elongated and nonelongated primers). These RMSD values are summarized in Table S2.

The average RMSD between the duplex regions of DNA in the free state and in complex with Taq polymerase across all modeled systems is approximately 2.4 Å. The largest structural deviation was observed for the L0/X4/S1 complex, with an RMSD of 3.3 Å. This pronounced difference is attributed to a marked widening of the minor groove in the protein-bound state. In this orientation, the modification effectively shields the nucleobases from solvent exposure and induces local stretching of the sugar–phosphate backbone. In contrast, the smallest RMSD values (i. e., the highest structural similarity between free and bound DNA) were found for modifications at positions X5 and X6 (Table S2). Furthermore, the RMSD between unmodified and modified DNA substrates – both in complex with Taq polymerase – averages ~1.75 Å. Notably, this deviation is smaller for modifications oriented toward the major groove, as these conformers minimize direct contacts with the protein.

The average RMSD which computed across all rotamers and stereoisomers for the DNA duplex in complex with Taq polymerase is approximately 2.0 Å. Lower RMSD values are observed for systems in which the N-benzimidazole modification adopts a consistent spatial orientation. Structural analysis further reveals that, even in cases of pronounced interactions between the modification and protein residues, the overall architecture of the duplex region remains largely unperturbed. In general, the structure of a substrate with a modification largely depends on which regions of the protein it interacts with, which is determined by both the isomer and the conformer of the N-benzimidazole residue

The structural parameters of the investigated nucleic acid substrates are predominantly characteristic of B-form DNA. However, localized deviations from ideal B-form DNA are observed in the vicinity of the 3′-end of the primer and at the site of N-benzimidazole modification. In particular, for nonelongated model systems (L0), a pronounced increase in the Roll and Buckle parameters was detected for AT base pairs adjacent to the catalytic center. For both extended and unextended complexes, the propeller twist angle of these AT base pairs was consistently negative, a feature more typical for A-tract DNA than canonical B-DNA (Strahs, Schlick, 2000). The Inclination of base pairs relative to the helical axis increased the closer the N-benzimidazole modification was positioned to the catalytic center. In contrast, this deviation markedly decreased in complexes with an elongated duplex region (L1–L5). Notably, the average Twist value across all systems remained approximately 34°, independent of duplex length or the presence and position of the modification. This constancy in Twist suggests that the helical packing density of the DNA duplex is largely preserved

In all studied complexes, a significant widening of the DNA minor groove (defined as the distance between phosphorus atoms on opposite strands) was observed in the region adjacent to the catalytic center, reaching 15–18 Å. In modified complexes, this widening increased further with the length of the duplex region (i. e., in L1–L5 systems), which corresponds to the progressive displacement of the modification away from the 3′-end of the primer. In contrast, native (unmodified) complexes exhibited a much smaller degree of minor groove width increase. No clear correlation was found between the structural parameters of the nucleic acid substrate and the specific spatial orientation of the modification. This suggests that the position of the modification relative to the 3′-primer terminus dominates its impact on global DNA conformation within the polymerase complex.

Analysis of sugar pucker conformations in the DNA duplex reveals that, in most cases, deoxyribose adopts the C2′-endo conformation which is characteristic of canonical B-form DNA. However, near the 3′-end of the primer, specific nucleotides, particularly those adjacent to the catalytic site, exhibit C1′-exo or O4′-endo sugar puckers. These non-canonical sugar conformations are indicative of local structural strain and are commonly associated with the catalytically active state of DNA polymerases.

The presence of the modification in the DNA strand within the Taq polymerase complex caused significant deviation from canonical planar base pairing only in the case of terminal and penultimate base pairs when the modification was located at the first or second position of the primer. Structural analysis shows that the modification does not affect the nature of base pairing: Watson–Crick pairs with standard hydrogen bond lengths are formed, except for the terminal base pairs – a finding previously observed both experimentally and in MD simulations (Nonin et al., 1995; Zgarbová et al., 2014). Thus, the modification at the first internucleotide phosphate residue exerted the greatest influence on the local DNA structure within the polymerase complex. Overall, the presence of the modification does not significantly alter the DNA structure, either in free duplexes or in the enzyme–substrate complex.

An analysis of the N-benzimidazole group orientation within the DNA duplex was performed for both the free state and the protein-bound complex. This was done by examining the dihedral angle around the P–N bond, defined by the nonbridging phosphate oxygen (OP1 for the Rp isomer and OP2 for the Sp isomer), the phosphorus atom, the nitrogen atom, and the carbon atom of the benzoazole ring. The analysis revealed considerable flexibility of the modified residue and the possibility of interconversion between rotameric states (Fig. S8).

**Fig. 4.**
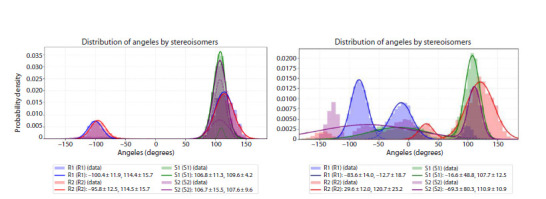
Dihedral angle values of the P–N bond in the phosphoramidate linkage for rotamers 1 and 2 along MD trajectories of free DNA (left) and DNA in complex with the protein (right), aggregated across all studied models.

Population analysis of the dihedral angles along the MD trajectories shows that, for both elongated and nonelongated systems, free DNA exhibits generally similar conformational preferences (Fig. S8, S9). The data indicate that the Rp isomer of the modified residue is predominantly oriented toward the minor groove, whereas the Sp isomer preferentially points toward the major groove, corresponding to a dihedral angle of approximately +100°. In some cases, the modification flips away from the duplex, corresponding to an angle of about –100° (rotamers R1 and S2). The lower population of this outward orientation is attributed to the hydrophobic nature of the benzimidazole group, which tends to minimize solvent exposure by interacting with the DNA strands. In most cases, the distributions for the two stereoisomers are qualitatively similar: when two peaks are present for one isomer, they are typically also observed for the other. Differences in peak amplitudes suggest that the conformational space for the modification is not fully sampled within the 50-ns trajectory of each individual model. However, when the angular probability distributions are aggregated across all modification positions for each stereoisomer, the average dihedral angles for rotamers 1 and 2 of each isomer nearly coincide (Fig. 4), indicating consistent conformational preferences irrespective of modification position.

In the protein-bound complex, the orientation of the modification undergoes significant changes compared to free DNA (Fig. 4). The plots of dihedral angle values and their probability distributions (Fig. 4, S9, and S10) show that, along the MD trajectories, angles are observed not only between the two main peaks characteristic of free DNA (+100° and –100°), but also shifted beyond these values to larger absolute magnitudes. This indicates substantial interactions between the modified residue and the protein, which constrain and redirect the conformational preferences of the N-benzimidazole group relative to its behavior in the unbound state

Comparison of the average probability distributions for different stereoisomers in the complexes shows that they differ significantly both from each other across modification positions and from the distributions observed for free DNA

(Fig. 4, S8, and S10). Notably, the probability distributions for rotamers R1 and R2 are markedly distinct. The main peak for R1 is located around –80°, corresponding to an orientation of the modification toward the major groove (i. e., away from the DNA helix). This is attributed to the fact that, in the polymerase complex, the native phosphate backbone is tightly coordinated by specific amino acid residues; consequently, the bulkier phosphoramidate modification is sterically expelled from the minor groove. In contrast, the primary peak for R2 appears near +100°, indicating that the modification is directed into the minor groove. For the S1 rotamer, the dominant angle is +100°, but the modification is oriented toward the major groove – a consequence of the opposite stereochemistry at the phosphorus center compared to the Rp series. The S2 rotamer exhibits a markedly different behavior: its probability distribution shows multiple peaks of comparable amplitude spread across nearly the entire angular range, indicating that the modification can adopt diverse spatial orientations depending on its position in the primer chain (Xj). This conformational heterogeneity is driven by specific, position-dependent interactions with the protein environment

It should be noted that, for all examined stereoisomers, a distinct peak appears around 0° (Fig. 4), corresponding to an orientation in which the modification points away from the DNA helix. In this conformation, one of the amino groups of the five-membered ring of the N-benzimidazole moiety forms a hydrogen bond with the non-bridging oxygen atom of the adjacent phosphate group. The absence of such orientations in free DNA indicates that this conformation is specifically stabilized by additional interactions with the protein, highlighting the role of the polymerase in shaping the conformational landscape of the modified backbone

*Analysis of interactions of modification with Taq polymerase*

**Fig. 5.**
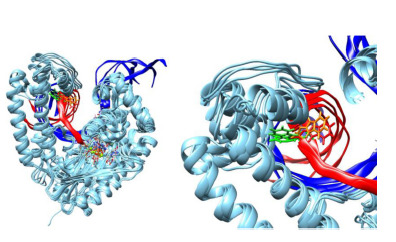
Structural comparison of the L0/X4 complexes: overall view (left) and close-up of the modification interaction region with the thumb domain of the enzyme (right). Taq DNA polymerase is shown in blue, the DNA template strand in blue, and the primer in red. The modified N-benzoazole groups are displayed as atomic models, with Sp isomers colored red and orange, and Rp isomers in light and dark green.

A hierarchical cluster analysis of the last 50 ns of each MD trajectory was performed to identify the most representative structures. The spatial arrangement of the N-benzoazole groups relative to the polymerase active site was examined, and the number of protein atoms in contact with the modification was quantified. Contact maps between the modification and Taq polymerase were also generated. All amino acid residues with at least one atom located within 3 Å of the modified phosphate group were considered to be in direct interaction with the modification (Tables S3 and S4). The DNA duplex region that engages with Taq polymerase spans 5–8 base pairs, and approximately 40 amino acid residues participate in this interaction. These residues are involved in nucleic acid recognition, substrate stabilization, and catalysis (Eom et al., 1996; Li Y. et al., 1998).Analysis of contacts between the phosphoramidate N-benzimidazole moiety and Taq polymerase revealed several key patterns. First, in the complexes L0/X1/R1, L1/X2/R2, L0/X3/R2, L2/X3/R1, and L4/X5/R2, the N-benzimidazole group was accommodated within protein pockets. Moreover, for the fourth modification position (X4) with the R stereoisomer, both rotamers (X4/R1 and X4/R2) occupied a pocket, forming stable interactions between the modification’s electronegative atoms and the protein’s positively charged arginine residues (Fig. 5).

Overall, modifications at positions 1–5 form an extensive network of hydrogen bonds and van der Waals contacts with the protein, whereas interactions for the 6th position are considerably weaker. Stereochemistry also strongly influences the binding mode: Sp stereoisomers preferentially interact with positively charged residues, while Rp stereoisomers more frequently engage in contacts with hydrophobic amino acids. Sp isomers are often oriented toward the major groove, effectively shielding the heterocyclic bases of the duplex from solvent exposure. In contrast, Rp isomers are predominantly directed away from the DNA and toward the protein surface. The presence of the modification frequently disrupts the regular nucleic acid structure due to interactions of the N-benzimidazole group with protein pockets, which induce strain in the sugar–phosphate backbone. Introduction of the modification at the first or second position of the primer leads to significant distortion of the terminal and penultimate base pairs. Moreover, in the complexes L1/X2/S2, L0/X3/R1, and L0/X2/R1, disruption of Watson–Crick base pairing near the modification site is observed.

According to the literature data, residue Arg660 from the fingers domain coordinates the phosphate group at the first position of the primer from the 3′-end, Arg587 from the palm domain coordinates the second internucleotide phosphate, and Arg536 from the thumb domain interacts with the fourth phosphate (Vinogradova, Pyshnyi, 2010). The presence of the N-benzimidazole modification is expected to neutralize the negative charge of the phosphate group and introduce steric hindrance that impedes coordination of the phosphate by arginine residues, which should reduce the catalytic rate. However, structural analysis shows that, in the case of Sp isomers at positions 2 and 4, the non-bridging oxygen atom of the phosphate moiety is still coordinated by Arg536 for both rotamers. Similarly, for Rp isomers with the modification at positions 1 or 2, at least one rotamer retains coordination of the phosphate oxygen by the corresponding arginine residue.

Both rotamers of the Rp isomer at the fourth position are accommodated within a hydrophobic pocket of the thumb domain, whereas the Sp isomer shows minimal interaction with the protein. As a result, the Rp-modified phosphate group impedes translocation of the polymerase to the next position along the DNA strand, which is required for incorporation of the subsequent nucleotide onto the primer. This steric and dynamic blockage most likely explains the accumulation of incomplete elongation products observed experimentally when the modification is located at the fourth position.

We have previously shown (Golyshev et al., 2025) that in primer elongation experiments with Taq DNA polymerase using primers bearing the N-benzimidazole modification, incorporation of the modification at the second position results in the smallest reduction in elongation efficiency for perfectly matched complexes. This correlates with the lowest number of contacts observed between the modification and the protein among the first three internucleotide phosphate positions. Furthermore, in all perfectly matched modified complexes, a distinct band corresponding to a partially extended primer, with the modification located at the 4th position from the 3′- end, was clearly observed. This effect is most pronounced for primers carrying modifications at the 1st and 3rd positions. These experimental observations correlate well with structural data showing that both rotamers of the R stereoisomer at position 4 (X4/R1 and X4/R2) are accommodated within a protein pocket and form stable interactions with the enzyme (Fig. 5).Thus, steric interactions of Rp isomers with protein pockets can slow down – or, as in the case of the fourth modification position, block – the translocation of Taq DNA polymerase along the substrate. This is experimentally confirmed by the reduced polymerization rate and the appearance of abortive elongation products of the modified primer containing the phosphoramidate N-benzimidazole group

*Substrate–polymerase interaction energy*

The interactions described in the previous section are reflected in the binding energetics between the enzyme and its substrate. Therefore, we calculated the interaction energy between the nucleic acid substrate and Taq polymerase using the Molecular Mechanics/Generalized Born Surface Area (MM/GBSA) calculation method, based solely on the MD trajectory of the protein–DNA complex. To minimize fluctuations in the computed free energy arising from the high flexibility of the single-stranded template overhang, only the duplex region of the nucleic acid substrate was included in the energy calculations. The energies of the DNA, protein, their complex, and the resulting binding (complexation) energies are reported in Tables S5 and S6. Analysis of the interaction energies between the modified nucleic acid substrates and Taq polymerase revealed the following trends: 1) for native (unmodified) complexes, the binding energy (in absolute value) increased with duplex length, reflecting stronger stabilization of longer primer–template hybrids within the polymerase active site; 2) in contrast, no clear correlation was observed between binding energy and duplex length for modified complexes; 3) notably, modifications at the 5th and 6th internucleotide phosphate positions exhibited weaker binding compared to all other model systems, which correlates with the reduced number of contacts between DNA and the protein observed in these cases.

In the case of nonelongated model systems (L0), which have the shortest duplex region, the complexation energy was, on average, significantly lower (~ –200 kcal/mol) than that of extended complexes (~ –180 kcal/mol). For the majority of complexes, S stereoisomers exhibited more favorable (i. e., more negative) binding energies compared to their Rp counterparts. This is likely due to the greater accessibility of the non-bridging oxygen atom of the modified phosphate group in the Sp configuration, facilitating its coordination by protein residues. Among the two rotamers, the S1 conformation – in which the N-benzimidazole group is oriented toward the major groove – consistently displayed the most favorable binding energy, as this orientation leaves the non-bridging phosphate oxygen exposed for interaction with amino acid side chains. It should be noted, no direct correlation was found between the number of protein atoms in proximity to the modification (Table S3) and the computed binding energy. However, the strongest enzyme–substrate binding was observed for the complexes L0/X4/R1 and L0/X4/R2, in which the modification is buried within a protein pocket and engages with the largest number of amino acid residues (Table S4).

## Conclusion

In this work, we employed molecular simulation and analysis to investigate the structure, dynamics, and interaction energetics of DNA substrates containing a phosphoramidate N-benzimidazole group at various positions within the primer strand in complex with Taq DNA polymerase. We found that both the position of the modification near the 3′-end of the primer and its stereochemistry significantly influence interactions with the enzyme. Within the enzyme–substrate complex, two stable rotamers were identified for each phosphoramidate stereoisomer (Rp and Sp). Analysis of the stereochemical effects revealed that Rp isomers generally exhibit stronger interactions with the polymerase, with the most pronounced binding observed when the modification is located at the fourth internucleotide phosphate from the 3′-end of the primer. Structural analysis of both DNA and protein showed no major global rearrangements in either biopolymer upon modification. Structural perturbations induced by the Nbenzimidazole group were either minor or strictly localized. The greatest impact on local DNA conformation within the polymerase complex was observed for modifications at the first internucleotide phosphate position

These computational findings correlate well with experimental data on the processing of PABAO primers by Taq DNA polymerase. In particular, they explain: 1) the reduced rate of full-length product formation for modified primers, 2) the accumulation of incomplete elongation products when the modification is located at the fourth position from the 3′-end of the primer, and 3) the significant decrease in primer elongation efficiency upon modification at the first position (Chubarov et al., 2024; Golyshev et al., 2025).

The results of this study provide a molecular basis for understanding how the phosphoramidate N-benzimidazole group affects the elongation of PABAO primers. These insights will be instrumental in the rational design of PABAO structures for applications in molecular diagnostics using PCR-based methods. Furthermore, the pronounced differences in polymerase interaction efficiency between Rp and Sp isomers of PABAOs highlight the need to develop stereoselective synthesis methods for these oligonucleotides. Such approaches would enable precise control over the stereochemistry of the phosphoramidate linkage, thereby allowing fine-tuning of the biochemical and biophysical properties of phosphoramidate benzazole oligonucleotides for optimized performance in diagnostic assays

## Conflict of interest

The authors declare no conflict of interest.
